# Delay of Gratification, Delay Discounting and their Associations with Age, Episodic Future Thinking, and Future Time Perspective

**DOI:** 10.3389/fpsyg.2017.02304

**Published:** 2018-01-25

**Authors:** Lars M. Göllner, Nicola Ballhausen, Matthias Kliegel, Simon Forstmeier

**Affiliations:** ^1^Department of Education Studies and Psychology, University of Siegen, Siegen, Germany; ^2^Department of Psychology, University of Geneva, Geneva, Switzerland; ^3^Center of the Interdisciplinary Study of Gerontology and Vulnerability, University of Geneva, Geneva, Switzerland; ^4^Swiss National Center of Competences in Research LIVES-Overcoming Vulnerability: Life Course Perspectives, Geneva, Switzerland

**Keywords:** self-regulation, delay of gratification, delay discounting, life span, future time perspective, episodic future thinking

## Abstract

The delay of gratification (DoG) in children is widely investigated with an experimental procedure originally called the “marshmallow test,” whereas the studies on self-regulation (SR) in adolescents and adults usually use self-report questionnaires. Delay discounting (DD) measures simplify the DoG procedure and focus on monetary rewards. The aim of this study was to investigate age differences in DoG and DD from childhood to old age using a test that is suitable for both children and adults. Furthermore, investigations were conducted on the association between DoG/DD and two future orientation constructs [future time perspective (FTP) and episodic future thinking (EFT)] as well as age differences in these constructs. Participants from five age groups (9–14, 18–25, 35–55, 65–80, 80+) participated in the study (*N* = 96). While we found no age difference for DoG, DD was the lowest [i.e., self-control (SC) was the highest] in young/middle adults; however, it was the highest (i.e., SC was the lowest) in children and old/oldest adults. Furthermore, we found significant age differences for DD and FTP. As predicted, there were strong correlations between DoG and FTP and between DD and FTP, but not between DoG/DD and EFT. These results indicate that age differences in SR vary across the measures used. Individuals who generally think and act in a future-oriented manner have a stronger ability to delay gratification.

## Introduction

In our everyday life, we often face situations in which it becomes essential to exercise patience for achieving a certain goal or mastering a certain challenge. It implies that the desire for immediate pleasures must be relinquished to achieve a long-term goal or to get a specified reward in the future. For example, a smoker might suffer momentarily from nicotine withdrawal, but will experience a healthier lung, lower risk of cardiac infarction, and other advantages in the long term.

In this context, and in the social and cognitive development of every individual, the ability to self-regulate plays a major role. Self-regulation (SR) can be defined as the behavioral skill of self-managing environmental conditions and to be able to enact this skill in relevant contexts (Boekaerts et al., 2005). Research has already dealt intensively with SR in humans and its effects on daily lives. Mischel et al. (1972) developed, in the context of their well-known “marshmallow experiment,” the delay of gratification (DoG) paradigm in order to operationalize SR on a behavioral level (see Mischel et al., 2011). Several other researchers support the opinion that DoG is a measure of SR (e.g., Mazur, 1987). DoG refers to the ability to delay an immediate reward to get a bigger reward at a certain point in the future. A considerable number of researches have dealt with DoG in children and how it can serve as an indicator for outcomes such as educational success, socioeconomic status, or future drug abuse (see Wulfert et al., 2002; Mischel et al., 2011). Besides, the ability to delay gratification in pre-school age impacts health in the old age (Moffitt et al., 2011) and can, for example, predict overweight (Seeyave et al., 2009) or rapid weight gain in early adolescence (Francis and Susman, 2009).

Long-term correlates of DoG and personality have also been found (Funder et al., 1983), and poor DoG has been shown to be a specific risk factor for externalizing disorders (Krueger et al., 1996). Furthermore, high DoG in childhood could be a protective factor against mental disorders like the borderline syndrome (Ayduk et al., 2008). In the field of social development, DoG ability in early childhood could serve as a predictor of pro-social behavior in the elementary school age (Paulus et al., 2015).

### A life-span perspective on delay of gratification

Existing studies on the subject have predominantly dealt with the long-term influence of DoG in childhood or adolescence on different variables later in life. However, there are important, but less well understood, factors in this context. A few of them include changes in DoG over the life span, whether there are differences in DoG between younger and older people, and what might be the associated developmental mechanisms. The current study addresses these issues in detail.

Green et al. (1994) were one of the first researchers to investigate age differences between children and younger and older adults with regards to their delay discounting (DD). DD refers to the phenomenon that the present value of delayed rewards reduces over time as a function of the delay interval. This construct is similar and highly correlated to DoG. Although similarities exist between DD and DoG, it is important to examine differences between the two constructs. The main disparity lies in the manner in which DoG and DD are measured. While a DoG test quantifies the behavior of the participant directly, the measurement of DD involves only hypothetical choices. Nevertheless, DoG and DD apply as the operationalization of impulsivity (Forstmeier et al., 2011), which in turn is seen as a central dimension of SR (e.g., Barkley, 2004; Raver et al., 2011).

Reynolds et al. (2002) describe DD as the tendency to devalue delayed rewards. For example, in DD, people would prefer getting $250 in 1 week instead of getting $200 immediately. But what happens, if the delay interval rises from 1 week to 3 months? Surely, some of the people would now rather choose the immediate reward over the delayed benefits (see Petry, 2001). The more often people choose the immediate reward over the delayed, the higher their discounting rate is marked. Therefore, it is important to mention that in the case of DD, higher discounting rates result in higher DD, which means lower SR. The results of Green et al. (1994) showed that the discounting rate for delayed rewards was highest for children and lowest for older adults, which indicates a developmental trend toward an increase in the ability to self-regulate over the life span. These results were supported by the findings of Harrison et al. (2002) who found a decline in discounting rates at least after middle age.

The results of later studies challenged these findings of a linear relationship between DD and age. Read and Read (2004) found in their study, comprising participants between 19 and 89, that both older and younger people discount more than middle-aged people, indicating a U-curve. Besides, lower discounting rates are linked to higher cognitive ability (Hirsh et al., 2008), whereas cognitive ability is lower in younger and elderly people (Rushton and Ankney, 1996). Therefore, literature shows contradictory results for the relationship between age and the tendency to devalue delayed rewards.

It was found that the marshmallow test of DoG and similar tests are inappropriate for application in adults because the type of reward (sweets) might not be attractive to many adults and the delay intervals are too short. Therefore, Forstmeier et al. (2011) developed and validated the *Delay of Gratification Test for Adults* (DoG-A). It includes four types of rewards that are meaningful to adults—, namely snacks, real money, hypothetical money, and magazines. In a sample of older adults, aged 60–94 years, Forstmeier et al. (2011) found a non-significant trend for persons in the age range of 60–69 years with the highest DoG values and participants older than 80 years with the lowest DoG values (Drobetz et al., 2012b). Taken together, the results of studies regarding SR and age are ambiguous but indicate toward an inverse U-curve for SR measured through DoG. Therefore, in the case of DoG, and contrary to DD, higher rates mean higher SR.

One plausible explanation for this inverse U-curve is that, in principle, young adults have more uncertainties in their life than older adults (Sozou and Seymour, 2003). They are unsure of their occupational career, potential life partner, and many other environmental circumstances. These uncertainties might be contributing toward their relatively low SR. In contrast, the middle-aged adults often have a more secured life situation with a completed education, permanent job, and organized family circumstances, and they can afford to make decisions from a long-term perspective. This sort of stability might account for their higher level of SR. Older adults have to face a different situation. They cannot be sure about the future because health problems become overwhelming at their age, and age-related physical and cognitive conditions may prevent them from living their lives as usually planned. These fluctuations in old age contribute toward comparatively lower SR (Trostel and Taylor, 2001). This hypothesis aligns with the idea of the *social selectivity theory* by Carstensen et al. (1999) too. According to the theory, individuals focus more on the present and less on the future when they realize that the rest of their life is limited (Drobetz et al., 2012b). Although most studies on age differences across the life span used DD measures, similar results can emerge with a DoG measure. This study is the first, to our knowledge, to use the same DoG measure for several age groups—from childhood to old age.

### Delay of gratification and future orientations

DoG and DD are based on futuristic perceptions and events. There are two future orientation constructs that seem to be particularly relevant to DoG and DD, namely episodic future thinking (EFT) and future time perspective (FTP). EFT is the ability to mentally travel into the future and, for example, imagine the feelings associated with a certain reward (Atance and O'Neill, 2005; Spreng and Levine, 2006; Schacter et al., 2008). Both, especially DoG and EFT, are based on imagining future scenarios or situations.

We also suggested that EFT has an important influence on DoG and DD because of the lower general cognitive capacity (Salthouse, 1991) and the special problems with imagining future scenarios in old age (e.g., Rendell et al., 2012). Furthermore, different cognitive functions (e.g., information processing) develop during childhood (Anderson, 2002).

In general, these studies on age differences regarding EFT draw the conclusion that younger adults are better time travelers. Rendell et al. (2012) showed that older adults, compared to young adults, perform worse in constructing atemporal and future scenarios as well as narrative that involved navigation. However, their results also indicate that the older adults had more problems in constructing future experiences than atemporal experiences, and thus, old age cripples an individual's ability to imagine the future (Spreng and Levine, 2006; Addis et al., 2008, 2010; Rendell et al., 2012).

Studies on EFT first found indications that there is a connection between better EFT and less DD, and a positive correlation to self-regulatory skills (Daniel et al., 2013, 2015). Furthermore, it makes sense that people with a better ability can imagine future events or situations, and thereby future rewards are also more likely to wait for gratification.

On the basis of these results, we predicted that individuals who have problems with mentally traveling into the future (low EFT) would also have difficulties in imagining a future reward and prefer immediate over long-term rewards (low DoG and high DD). That is why we also predicted an inverse U-shaped form of the EFT performance depending on age. Literature already showed this type of forms, testing the relationship between age and different cognitive functions (e.g., Cerella and Hale, 1994).

The second future orientation construct relevant to DoG is FTP. According to FTP, every individual exhibits a relatively stable differential dimension that determines the individual's perspective on time, and this perspective can be developed into future, present, or past orientation. This orientation is expressed in the individual's attitudes and behavior, and is among other things related to our identity, motivation, interpersonal interaction, and emotion (Webster, 2011). On the one hand, Webster already showed the positive influence of an FTP on different important psychological variables. On the other hand, he additionally created four different types of time perspective categories on the basis of his data analysis. These four categories are the *time expansive* category, with a balanced and intense time perspective of future and past (individuals with rather positive thoughts and feelings about the future and the past); the *time restrictive* category, with less interest for future or past events (contrary individuals with rather negative thoughts about the future and the past); the *reminiscers* category, with a perspective directed in the past (these individuals showed a preferred view toward the past); and the *futurist* category with a time perspective directed into the future (individuals rather thought about the future than the past).

FTP impacts a variety of important factors of our lives. Individuals with a perspective directed rather into the future show more motivation in achieving and studying (Shell and Husman, 2001), perform better on academic DoG (a measure for self-regulated learning, Bembenutty and Karabenick, 2004) and exhibit more successful learning behavior (e.g., Husman and Lens, 1999) as well as better financial behavior (Jacobs-Lawson and Hershey, 2005). Besides, higher work motivation (Seijts, 1998) and more responsible use of contraceptives (Burns and Dillon, 2005) are behavioral variables that are linked to a higher FTP. In addition, Daugherty and Brase (2010) found that a higher FTP predicted less tobacco, alcohol, and drug use, and in exchange predicted healthier behavior such as eating breakfast or wearing a safety belt.

To sum up, FTP is positively associated with a variety of self-regulatory behaviors and outcomes. Therefore, we predict that the ability to delay gratification is associated to a futuristic view. This owes to the fact that an individual has to wait for and imagine the bigger delayed reward that has a better potential over smaller immediate rewards. Hence, firstly, an FTP should stimulate thoughts about future rewards and its advantages and therefore, facilitate delaying gratification. As a second argument, DoG is a measure of motivational SR; furthermore, as discussed earlier, the research has demonstrated a correlation between FTP and other measures of SR or motivation.

### Goals of the present study

This study had three aims. The first aim was to delineate possible age differences in DoG and DD across the life span. The reviewed literature indicates an inverse U-shaped function of DoG, with young and older people performing worse on DoG when compared with the middle-aged people, and a U-shaped form for DD.

The second aim was to investigate the association between DoG/DD and two future orientation constructs—*future time perspective* and *episodic future thinking*. We predicted a positive correlation between DoG/DD and EFT, and DoG/DD and FTP. In contrast, we expected that there is no significant correlation between DoG/DD and past time perspective. Individuals who are high in FTP are expected to have higher DoG values (and lowest DD values) than individuals low in FTP.

The third aim was to investigate age differences with regards to the future orientation constructs (FTP and EFT) and different types of time perspectives.

## Methods

### Participants

A total of 96 participants, aged between 9 and 101 years, were enrolled in this study. The participants from five age groups (9–14, 18–25, 35–55, 65–80, 80+) were recruited in the regions of Zurich and Lucerne, Switzerland. Recruitment sources included the participant server of the Dept. of Psychology of the University of Zurich, the database of the authors, and several clubs for children (e.g., a soccer club). The participation was voluntarily upon receiving an oral or written invitation. In the case of participants below 18 years, one of the parents also had to give the consent. The participants were offered one reward as a motivation for participating in the study. These rewards included either 20 CHF (approximately 20 USD), 1.5 participant hours for their course credits (in the case of psychology students), or a voucher for an activity (visit to cinema for adolescents or toys' shop for children).

The participants were screened for cognitive impairment and depression, and the individuals with cognitive impairment or elevated depression scores above the critical cutoff were not included in the study.

### Assessment of delay of gratification and delay discounting

#### Delay of gratification test

The *Delay of Gratification Test for Adults* (Forstmeier et al., 2011), a behavioral measure of motivational SR, was presented in the form of a board game. The test was originally developed for use in adults, but the simple tasks can be understood and performed from the age of eight, as pilot data have shown. This test included four decision tasks, and every decision task involved different types of rewards (e.g., snacks, hypothetical money, real money, and magazines). The snacks' task was adapted from Knolle-Veentjer et al. (2008), and the real money task was adapted from Wulfert et al. (2002). The experimenter and each participant took turns in moving a counter through the streets of a fictitious city. In the game, at each field on the board, the player draws a card and makes a decision in a fictitious shop.

The participants were not informed about the real aim of the test (measuring DoG) and the experimenter gave an impression that the game aimed at measuring their preferences and interests. Filler items were spaced between the decision tasks to gauge the participant's preferences about products available in the shop. It included queries such as: “There are black and red pullovers on sale. Do you like the black or the red pullover better?”

The four delay of gratification tasks were as follows (see Forstmeier et al., 2011, for concrete instructions):

Snacks: In eight trials, the participants were supposed to decide between 1 piece of snack (depending on the preferences that they specified before their visit) or 2 pieces (2 h after the experiment). As per the experimental design, the participants were supposed to choose two favored snacks from a list that they received a few days before the experiment was realized. This ensured that each participant was presented with an attractive incentive.

Hypothetical money: In further eight money task trials, each participant was supposed to choose between an immediate and a delayed hypothetical money reward. The immediate amount varied between 6 and 9.50 CHF in steps of 0.50 CHF (presented in the order 9.50, 6.00, 6.50, 9.00, 8.50, 7.00, 7.50, and 8.00), whereas the delayed amount was always 10 CHF. The experimenter asked, “Imagine that a friend of yours has won some money in the lottery. He or she wants to give you some money as a present. But you have to choose between CHF 6 now and CHF 10 in 1 month…”

Real money: In the real money task, the experimenter offered a chance to decide between an immediate reward of 8 CHF and a delayed reward of 10 CHF, which the participants were entitled to receive in 1 month's time. It was plausibly explained that the participants would receive individual feedback on task results along with the 10 CHF banknote within a month's time. The money was placed on a table and was visible to both the experimenter and participant.

Magazine: As in the case of snacks, a few days before the experimental session, participants were provided with a list of magazines. The participants were asked to pick one favorite magazine from the list, and the chosen magazine was purchased for the session. In the testing session, after the pawn moved across a certain field, the experimenter placed the magazine on the table and explained that the participant can choose either getting the exhibited magazine immediately or receiving two magazines (the exhibited magazine and the next issue) along with feedback on the study results via mail in 1 month.

Altogether there were 18 trials in the course of the game—eight for the snack, eight for the hypothetical money, one for the real money, and one for the magazines. Subscores for every type of reward were calculated. In the case of snacks and hypothetical money, the number of delayed rewards defined the final score (0–8). In the case of real money and magazines, the participants only had two choices (immediate vs. delayed reward), which implies that the score was dichotomous (0 vs. 1). The composite DoG score was drawn by first dichotomizing the two continuous variables (snacks and hypothetical money), with the scale mid-point as cutoff (0–4 vs. 5–8), and calculating the sum of the four subscores.

Forstmeier et al. (2011) reported the criterion validity with the help of the calculated bivariate correlations between the subscores and different variables, which are known as indicators for SR. The correlations with DD were the highest (*r* = −0.46, *p* < 0.01).

#### Delay discounting questionnaire

Delay discounting was measured using the 27-item *delay discounting questionnaire* (DDQ) by Kirby et al. (1999) in its German version (Forstmeier and Maercker, 2011). The DDQ is a well-established test for measuring DD for adults and also for children (Wilson et al., 2011; Daniel et al., 2015). The questionnaire provided an option to each participant to choose between either a smaller immediate amount of money and a larger financial reward in future. The instruction was to imagine receiving a monetary reward, which implied the non-employment of real money in the test. The delay interval differed across the items. The first item in the questionnaire was as follows: “Would you prefer CHF 68 today or CHF 69 in 92 days?” This statement was in contrast to the eleventh item: “Would you prefer CHF 14 today or CHF 38 in 7 days?” The range of the delay was between *R* = 7–214 days (*M* = 74.10) while the money amounts of the delayed rewards differed between *R* = 32–107 CHF (*M* = 69.44). Discounting rates were estimated on the basis of a pattern of 27 choices. These 27 choices were divided into 3 magnitude categories: small (CHF 32–44), medium (63–76), and large (CHF 95–107) (see Forstmeier and Maercker, 2011, p. 122). They were first estimated separately for each magnitude category and then averaged as the geometric mean to calculate a global discounting rate *k*. We used the geometric midpoint to avoid underweighting the smaller of the two parameters (see Kirby and Maraković, 1996, p. 102). Following Mazur (1987), a hyperbolic decay function describes the discounting curves the best:

$$\begin{array}{l} V=\frac{A}{\;1+kD} \end{array}$$

*V* describes the present value of the delayed reward *A* at delay *D*, while *k* symbolizes a free parameter, which reflects the discounting rate (see Forstmeier and Maercker, 2011, p. 122). *k* increases as an individual's preference for immediate rewards increases. Therefore, a higher *k* can be interpreted as higher impulsiveness or a lower level of SR.

The reliability (consistency) of the German DDQ, which is measured as the percentage of accordance between the individual's decision and the computed discount rate, has been shown to be very high, i.e., 98.3%, in the present sample. The bivariate correlations with measures of DoG (*r* = −0.46, *p* < 0.01) and impulsivity (*r* = 0.21, *p* < 0.05) confirmed the construct validity of the German DDQ (Forstmeier and Maercker, 2011).

### Assessment of future orientations

#### Episodic future thinking

The approach of Hassabis et al. (2007) and Rendell et al. (2012), which includes an imagination task, was adopted in the current study to investigate the ability of EFT. The participants got the instruction to imagine and describe four specified scenarios to the experimenter. The main purpose of the task in our study was to find the quality and coherence of imagination of future events. In contrast to Rendell et al. (2012), only two atemporal and two future scenarios were used in the study. The spoken words of the participants were recorded, transcribed, and coded.

The participants imagined and provided detailed specifications of each of the four scenarios that were introduced by the experimenter. They got the instruction not to use a previously experienced or familiar situation, but to imagine a new scenario. For example, the instruction for the beach scenario (atemporal) was as follows: “Imagine you are lying at a deserted beach with white sands in a beautiful tropical bay. Now, please give a detailed description of your experience and surroundings, using all your senses and everything you can see, hear, and feel” (see Hassabis et al., 2007). Atemporal scenarios were used to minimize the level of difficulty and the relationship to the innate ability of creative thinking. During the future scenario tasks, the participants had to envision themselves within a specific future situation and were asked to picture it as if they undergo the scenario at the present moment (“Imagine how you will spend next Christmas,” see Rendell et al., 2012). Therefore, the atemporal scenarios as well as the future scenarios tested the individual's imagination vividness. However, in the future condition, participants additionally had to mentally travel into the future and imagine a futuristic scenario, which extends the required skill set.

The participants were asked to complete a questionnaire upon the completion of each scenario. This questionnaire comprised five questions (e.g., “How difficult was the task?”). In the final step, the participants were required to confirm or deny 12 statements concerning the imagining task for each of the four scenarios (e.g., “I saw the scene in color”). On the one hand, the scoring involved the individual's own evaluation of the imagined scenarios; and on the other hand, it comprised a rating on content and quality by an independent rater who was unaware of the participant's personal data. The result of the full scoring procedure is referred to as “experience index,” which measures the level of detail in an individual's imagination. The index is composed of four subscales.

Firstly, after every imagination task, the participants were required to assess the feeling of presence and noticed salience through a questionnaire. Secondly, the participants rated 12 statements concerning the spatial coherence of the imagined scenario. Thirdly, the transcribed voice recordings were segmented into a set of statements by an independent rater. Following this process, each statement was classified as belonging to one of the following four content categories: spatial reference, entity presence, sensory description, and thought/emotion/action. Fourthly, an independent rater estimated the general quality of the imagination. With regards to each scenario, the rater scored for the strength of the feeling that the imagination of the participant created before his inner eye (for more details see Hassabis et al., 2007; Rendell et al., 2012). After analysis, every participant had a composite score, otherwise known as experience index, for every single scenario. The experience index ranged from 0 (not experienced at all) to 60 (extremely richly experienced). The score was calculated by summing up the four subscores. The performance of the participants was rated by calculating a mean for the two atemporal (EFT-A) scenarios and a mean for the two future scenarios (EFT-F, which is used to indicate the term *episodic future thinking* in this article).

#### Balanced time perspective scale (BTPS)

The time perspective of the participants was measured using the Balanced Time Perspective Scale by Webster (2011). It contains 28 items divided in 2 subscales, each comprising 14 items with the possibility to answer on a 6-point Likert scale. One subscale measures positive feelings related to the participant's past (e.g., “Remembering past accomplishment makes me feel good about myself”), and the second subscale is based on the future of the participants (e.g., “Planning for the future gives me a sense of direction in life”). According to Webster (2011), the selected items cover cognitive, emotional, and motivational dimensions of future (*future time perspective*, FTP) and past orientation (*past time perspective*, PTP*)*. Besides, like already mentioned in the introduction, each participant can be assigned to one of four groups, after median split of the two scores.

We built the time categories of the BTPS following Webster's original work (2011). Therefore, we performed a median split on the two subscales (past and future) regarding the scores of our sample independent of the age categories. The participants low in both, the past and future subscale, were assigned to the *time restrictive* category. Persons in this category are characterized as rather not thinking in long-term goals and disdaining past experience. Those participants low in the past subscale and high in the future subscale were assigned to the *futurist* category with a view rather directed into the future. The participants were assigned to the *reminiscers* category, if they had a low future subscore and a high past subscore. These persons orient themselves above all toward the past. Finally, the participants high in both subscales were assigned to the *time expansive* category. Being in this category means taking past experiences as well as future possibilities into account. During the collection of data, we made the experience that all age groups could understand and answer the questions appropriately.

### Other variables

#### Depression

The participants were screened for a possible depressive syndrome using the German depression test for children (DTK, Rossmann, 2008). This test was used for the youngest age group, while adult participants were tested through the German version of the Geriatric Depression Scale (GDS, Gauggel and Birkner, 1999). The DTK consists of 55 items and is a self-report measure of depressive symptoms for children above 9 years. The GDS consists of 15 questions and exhibits a dichotomous answer format (yes/no).

#### Verbal intelligence

The verbal intelligence of the participants was measured using the vocabulary test of the HAWIK-IV (Petermann and Petermann, 2008). While this test was employed for the youngest age group, the German vocabulary test was employed for adult participants (Schmidt and Metzler, 1992).

The German vocabulary test contains 42 items, presenting five words in a row. As per the test design, the participant had to differentiate between one real word and four tractions and had to mark the real word. The number of correct answers generates a raw score, which can be transformed into a standard score and interpreted with the help of a norm sample.

The vocabulary test of the HAWIK-IV is the German version of the Wechsler Intelligence Scale for Children (WISC). It includes 30 tasks, wherein a booklet is placed in front of the children on a table, and the children are asked the meaning of a word while they are reading that word in the booklet. The participants can score 0, 1, or 2 points in each task. The final score is the sum of scores achieved in each task.

### Procedure

The participants received oral and written information about the study's goals and procedures. They signed a consent form. The testing session, lasting for approximately 90 min, started with either the DoG or the EFT task. The study randomized the order of the DoG and the EFT to control the possible impact of EFT on the DoG performance over the entire sample. However, the study found no such effect, and hence, the order of the two tests was not considered during the analysis.

Later, a series of questionnaires was administered in the following order: delay discounting, FTP, depression, and demographic variables. Finally, cognitive tests were administered.

At the end of the testing session, the participants were orally debriefed and were handed a written explanation. In addition to a compensation for their time (either 20 CHF, 1.5 participant hours or a voucher for children), participants received a magazine, snacks, and 8 or 10 CHF as rewards associated to the DoG test.

### Data analysis

Data analysis was realized using IBM Statistics 23.0 for Mac, with a standard alpha of 0.05. Data screening showed that most of the reviewed variables were not normally distributed (DoG1, DoG2^1^, DD, FTP, PTP, age, depression-adults, verbal intelligence-adults, depression-overall, verbal intelligence-overall, income, education). Normally distributed variables included EFT-F, EFT-A, depression-children, and verbal intelligence-children.

We computed the correlative relationships (Kendall's Tau, τ) between all the variables. The study also investigated between-group differences of the sample characteristics and main study variables through analysis of variance (ANOVA) and the Kruskal-Wallis Test. The *least significant difference test* (LSD) was also employed to test the significance of potential *post-hoc* between-group effects. Furthermore, we tested linear and non-linear regression models and their fit regarding the main study variables. We hypothesized that a non-linear quadratic function (in the shape of an inverse U for DoG and in the shape of a normal U for DD) would be the best fit for the data.

Additionally, the study measured control variables that had been shown to be relevant in other studies investigating DoG or DD concerning age differences or similar relationships—sex (Jacobsen et al., 1997; Romer et al., 2010; Forstmeier et al., 2011), depression (Read and Read, 2004; Gawrilow et al., 2011) and verbal intelligence (Gawrilow et al., 2011; Drobetz et al., 2012b). Besides, a *z*-transformed value, which is suitable for every age group and test version, was computed for comparing the scores of the different variables that were collected with the help of different test versions for younger and older participants.

## Results

### Age differences

#### Sample characteristics

The descriptive sample characteristics and main study variables are presented in Table 1. Differences between the groups were not found for sex [$\chi_{\left(4,\;96\right)}^{2}$ = 3.50, *p* = 0.478], depression [$\chi_{\left(4,\;95\right)}^{2}$ = 6.44, *p* = 0.169], and verbal intelligence [$\chi_{\left(4,\;96\right)}^{2}$ = 2.71, *p* = 0.608]. The groups differed, and this difference increased for education [$\chi_{\left(4,\;96\right)}^{2}$ = 50.53, *p* < 0.001] and income [$\chi_{\left(4,\;92\right)}^{2}$ = 73.97, *p* < 0.001].

**Table 1:** Differences between age groups.

	**Age groups**					**ANOVA or Kruskal-Wallis Test^b^**	**ANCOVA**		
						***F or χ*^2^**	***p^c^***	***F***	***p^d^***
	9 – 14 years	18 – 25 years	35 – 55 years	65 – 80 years	85+ years				
	(*n* = 18)^a^	(*n* = 25)^a^	(*n* = 18)^a^	(*n* = 25)^a^	(*n* = 15)^a^				
**MAIN STUDY VARIABLES**									
Delay of gratification1	3.0 (1.2)	2.7 (1.1)	2.9 (1.2)	2.1 (1.2)	2.7 (1.2)	7.87	0.097	–	–
Delay of gratification2	2.78 (1.26)	2.48 (1.08)	2.55 (1.15)	1.88 (1.23)	2.30 (1.16)	6.98	0.137		
Delay discounting	−5.3 (−1.5)	−5.9 (−1.7)	−6.0 (1.7)	−4.9 (−1.5)	−4.6 (−1.7)	12.47^*^	0.014	–	–
Episodic future thinking, future	28.8 (7.3)	35.9 (8.7)	43.5 (8.4)	34.0 (8.8)	35.3 (8.1)	7.16^***^	<0.001	8.24^***^	<0.001
Episodic future thinking, atemporal	30.8 (8.6)	36.2. (8.7)	43.3 (7.6)	35.4 (10.5)	36.2 (8.0)	4.67^**^	0.002	5.01^***^	<0.001
Future time perspective (BTPS)	64.2 (8.8)	64.0 (11.2)	64.1 (11.8)	58.2 (11.8)	53.3 (17.4)	8.60	0.072	–	–
Past time perspective (BTPS)	61.6 (8.0)	52.4 (9.6)	56.3 (14.5)	62.7 (7.6)	67.0 (9.5)	5.68^***^	<0.001	7.19^***^	<0.001
**ADDITIONAL SAMPLE CHARACTERISTICS**									
Age, years	11.3 (1.36)	21.8 (1.8)	42.3 (5.8)	73.4 (3.5)	90.0 (5.2)	90.40^***^	<0.001	–	–
Sex, % Female	44.4	68.0	66.67	52.0	50.0	3.50	0.478	–	–
Education, years	5.8 (1.5)	17.0 (2.5)	15.8 (3.6)	14.5 (3.5)	15.2 (4.1)	50.53^***^	<0.001	–	–
Income	22.4 (11.9)	1065.2	5330.6	6084.1	6800.0	73.97^***^	<0.001	–	–
		(630.3)	(2884.8)	(3202.4)	(2307.1)				
Depression self–report (GDS), adults	–	3.3 (2.6)	2.2 (2.7)	1.8 (2.0)	2.9 (2.1)	6.22		–	–
Depression self–report (DTK), children	15.0 (8.4)	–	–	–	–	–		–	–
Depression self–report (overall)	0.0 (1.0)	0.31 (1.05)	−0.13 (1.1)	−0.30 (0.78)	0.17 (1.0)	6.44	0.169	–	–
Verbal intelligence (WST), adults	–	33.48 (2.2)	32.3 (5.1)	43.4 (2.5)	32.5 (5.1)	2.67		–	–
Verbal intelligence (WST), children	47.4 (7.1)			–	–	–			
Verbal intelligence (overall)	0.0 (1.0)	−0.03 (0.61)	−0.30 (1.4)	0.28 (0.70)	−0.25 (0.14)	2.71	0.608		

*All three analyses respectively enclosed five different age groups (9–14; 18–25; 35–55; 65–80; 85+)*.

*Abbreviations: BTPS, Balanced Time Perspective Scale; DTK, Depressionstest für Kinder (Depression Test for children); GDS, Geriatric Depression Scale; WST, Wortschatztest (Vocabulary Test); overall, standardized z-scores over the whole sample*.

a *Unless otherwise specified, the data represent means (±SD)*.

b *Variables analyzed via ANOVA: Episodic Future Thinking, Future; Episodic Future Thinking, Atemporal; Past Time Perspective; Depression, children, Verbal Intelligence, children. Variables analyzed via Kruskal-Wallis-Test: Delay of Gratification1 (built with the cutoff between 4 and 5 on the snack scale); Delay of Gratification2, (built with the cutoff between 6 and 7 on the snack scale); Delay Discounting; Future Time Perspective; Age; Sex, Education; Income; Depression, adults; Verbal Intelligence, adults; Depression, overall; Verbal intelligence, overall*.

c P-value ANOVA and Kruskal-Wallis-Test. ^*^p < 0.05, ^**^p < 0.01, ^***^p < 0.001.

d P-value ANCOVA. ^*^p < 0.05, ^**^p < 0.01, ^***^p < 0.001.

#### Group differences

**Figure 1:**
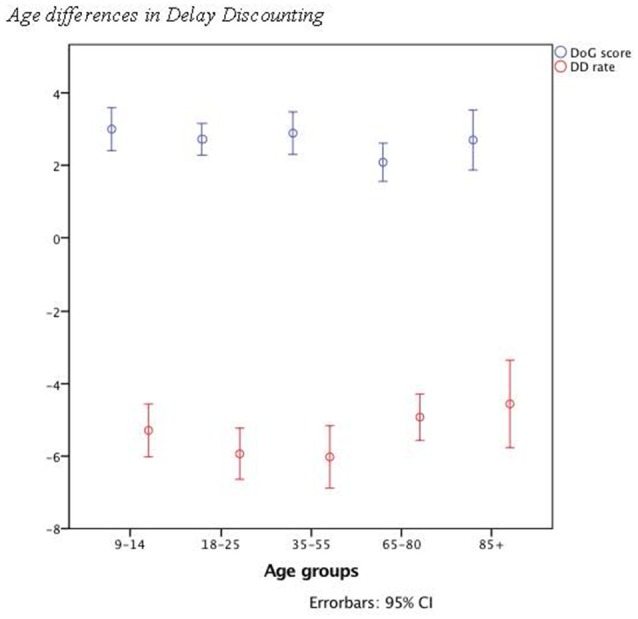
Age differences in Delay Discounting. This figure shows the differences in Delay Discounting and Delay of Gratification behavior between the five age groups. As expected, we can see a U-shaped form. Especially, the youngest and oldest participants showed higher levels of Delay Discounting and therefore a more impulsive behavior. These was no significant differences of DoG.

While the study revealed significant age group difference for DD [$\chi_{\left(4,\;96\right)}^{2}$ = 12.47, *p* = 0.014], there was only a marginal trend for DoG1 [$\chi_{\left(4,\;95\right)}^{2}$ = 7.87, *p* = 0.097] and no effect for DoG2 [$\chi_{\left(4,\;95\right)}^{2}$ = 6.98, *p* = 0.137; see Table 1]. Therefore, DD was lowest (i.e., SR was highest) in young and middle age adults, but highest (i.e., SR lowest) in children and older adults (see Figure 1). To detect, which groups differed, we used U-tests with an adjusted alpha-level (α = 0.005). The results showed only a significant difference for the comparison of the middle-aged group and the oldest group (*z* = −2.91, *p* < 0.005). Analysis of covariance (ANCOVAs) was performed (in case of the normally distributed variables) to exclude a systematic influence of the variables sex, depression, and verbal intelligence (see Table 1). The results did not show different patterns after eliminating the influence of the mentioned covariates.

We found an age difference for the future scale of EFT [EFT-F; *F*_(4, 96)_ = 7.16, *p* < 0.001] as well as the atemporal scale of EFT [EFT-A; *F*_(4, 96)_ = 4.67, *p* = 0.002]. While the results only showed a small trend for age differences in FTP [FTP; $\chi_{\left(4,\;95\right)}^{2}$ = 8.60, *p* = 0.072], there were differences for past time perspective [PTP; *F*_(4, 95)_ = 5.68, *p* < 0.001]. When controlling for sex, verbal intelligence, and depression there were significant age difference for EFT-F [*F*_(4, 96)_ = 8.38, *p* ≤ 0.001], EFT-A [*F*_(4, 96)_ = 5.01, *p* < 0.001], and PTP [*F*_(4, 95)_ = 7.19, *p* = 0.001]. *Post-hoc* comparisons using *t*-tests with Bonferroni corrections indicated that the middle-aged group differed significantly from all other age groups except of the oldest (35–55 vs. 9–14: *M* = 14.66, *p* < 0.001; 35–55 vs. 18–25: *M* = 7.52, *p* < 0.05; 35–55 vs. 65–80: *M* = 9.46, *p* < 0.01).

#### Regressions

Besides, non-linear regression models were calculated to consider the inverse U-shaped or simple U-shaped forms of the hypothesized relationships between DoG1/DD/EFT-F and age. Therefore, the study used models based on a quadratic function to picture the hypothesized relationships. A non-significant fit of the regression model was found for the relationship between DoG1 and age [*F*_(2, 92)_ = 1.92, *p* = 0.15], with an *R*^2^ of 0.04. The fit of the quadratic regression model for the relationship between DD and age was significant [*F*_(2, 92)_ = 4.60, *p* = 0.013], with an *R*^2^ of 0.091. A linear regression was significant too, but with an *R*^2^ = 0.043, which means less explained variance, and therefore, a weaker fit of the model. However, we have to mention that both *R*^2^ did not differ significantly. After z-transforming the *R*^2^ of both models using Fisher's z-transformation, we compared the confidence intervals of both coefficients. The 95% CI [0.1, 0.5] of the quadratic model overlapped the 95% CI [0.01, 0.4] of the linear model. The overlapping of both CI means that the coefficients did not differ significantly.

Furthermore, a significant regression equation was found for the relationship between EFT-F and age [*F*_(2, 92)_ = 10.02, *p* < 0.001] with an *R*^2^ of 0.18. Again the model of the linear regression (*R*^2^ = 0.009) did not match the data as well as a model with quadratic function. The difference between both *R*^2^ was not significant (*z* = 1.54, *p* = 0.124).

The results for DoG2 did not differ seriously. The regression model based on a quadratic function did not fit the relationship between DoG2 and age significantly [*F*_(2, 94)_ = 2.12, *p* = 0.126], with an *R*^2^ of 0.04. We could only find a trend for the relationship between DoG2 and age based on a linear function [*F*_(1, 94)_ = 3.62, *p* = 0.06], with an *R*^2^ of 0.037.

Additionally, we computed a mediation analysis between DoG/DD and FTP with age as mediator and the z-scores verbal intelligence and depression as covariates using *Process* by Hayes and Scharkow (2013). We found a significant direct negative effect between DoG1 and FTP (*b* = 0.036, *p* = 0.016) and a non-significant indirect effect considering age as mediator, which means that the relationship between DoG1 and FTP is at least slightly influenced by age (CI [−0.0265, 0.0113]). The results of the mediation analyses between DD and FTP followed the same pattern. We included the same mediators and controls as for DoG1 (*b* = −0.037, *p* = 0.031; CI [−0.0222, 0.0063]). The results of DoG2 did not show different patterns than the results of DoG1.

### Correlations

In addition to the age differences, the study investigated the correlations (Kendall's Tau, τ) between the main study variables (see Table 2). DoG correlated, as expected, highly significantly and negatively with DD (*r*_τ_ = −0.394, *p* < 0.001). DoG1 also correlated highly significantly with FTP (*r*_τ_ = 0.263, *p* < 0.01), but not with PTP (*r*_τ_ = −0.028, *p* = 0.720), as hypothesized. Contrary to our expectations, DoG1 did not correlate with EFT. The same pattern was found for DD wherein a significant correlation existed with FTP (*r*_τ_ = −0.172, *p* < 0.05), but not with PTP and EFT. Furthermore, positive correlation was found between EFT-F and EFT-A (*r*_τ_ = 0.541, *p* < 0.001), and between FTP and EFT-F (*r*_τ_ = 0.155, *p* < 0.05).

**Table 2:** Correlations of delay of gratification, delay discounting, episodic future thinking, time perspective and age.

**Variable^a^**	**DoG**	**DD**	**EFT-F**	**EFT-A**	**FTP**	**PTP**	**Age**
Delay of gratification1 (*n* = 95)	–	−0.394^***^	−0.084	−0.072	0.263^**^	−0.028	−0.140
Delay of gratification2 (*n* = 95)	0.882^***^	−0.428^***^	−0.121	−0.136	0.221^**^	−0.041	−0.143
Delay discounting (*n* = 96)		–	−0.056	−0.026	−0.172^*^	0.102	0.134^b^
Episodic future thinking—future (*n* = 96)			–	0.541^***^	0.155^*^	0.008	0.139^*^
Episodic future thinking, atemporal (*n* = 96)				–	0.073	0.056	0.111
Future time perspective (*n* = 95)					–	0.112	−0.168^*^
Past time perspective (*n* = 95)						–	0.179^*^
Age (*n* = 96)							–

*DoG, Delay of Gratification1 (built with the cutoff between 4 and 5 on the snack scale); Delay of Gratification2 (built with the cutoff between 6 and 7 on the snack scale); DD, Delay Discounting; EFT-F, Episodic Future Thinking Future; EFT-A, Episodic Future Thinking Atemporal; FTP, Future Time Perspective; PTP, Past Time Perspective*.

a *Kendalls's τ*.

b *p-value = 0.060*.

* *p < 0.05*,

** *p < 0.01*,

*** *p < 0.001*.

We found similar results for the correlations of DoG2 as for DoG1. Therefore, we found a significant negative correlation to DD (*r*_τ_ = −0.428, *p* < 0.001) and a positive relationship to FTP (*r*_τ_ = 0.221, *p* < 0.01) but no significant correlation to PTP (*r*_τ_ = −0.041, *p* = 0.601). As for DoG1, we could not find a significant correlation to EFT-F as hypothesized (*r*_τ_ = −0.121, *p* = 0.114).

The correlations with age as continuous variable showed several significant linear relationships. Age and FTP were negatively correlated (*r*_τ_ = −0.168, *p* < 0.05), whereas age and PTP were positively associated (*r*_τ_ = 0.179, *p* < 0.01). There is a strong trend for a rise in DD with age (*r*_τ_ = 0.134, *p* = 0.060) and a small negative trend for DoG1 and age (*r*_τ_ = −0.140*, p* = 0.072). Furthermore, we found a positive relationship between age and EFT-F (*r*_τ_ = 0.139, *p* = 0.048).

Additionally, the alternative results using DoG2 showed a trend for a negative relationship between DoG2 and age (*r*_τ_ = −0.143, *p* = 0.065).

### Differences between time perspective categories

After median split of the two dimensions future and past orientation, 29 subjects were classified as *time restrictive*, 19 as *futurists*, 22 as *reminiscers*, and 25 as *time expansive*. Analysis showed that the groups differed significantly for DoG1 [$\chi_{\left(3,\;\text{N}\;=\;94\right)}^{2}$ = 17.61, *p* = 0.001], EFT-F [*F*_(4, 94)_ = 3.07, *p* = 0.032], and only with a small trend for DD [$\chi_{\left(3,\;\text{N}\;=\;95\right)}^{2}$ = 6.96, *p* = 0.073]. The categories did not differ significantly for EFT-A [*F*_(4, 94)_ = 2.01, *p* = 0.118]. Descriptive analysis showed that the *futurists* were best at delaying gratification, followed by the persons in the *time expansive* category, the *reminiscers*, and subjects in the *time restrictive* category. The alternative analysis with DoG2 did not show different patterns and only slightly different results (see Table 3). Again, we used U-tests with an adjusted alpha level (α = 0.008) to detect *post-hoc* groups differences. We could find significant *post hoc* differences for DoG1 between *time expansive* and *time restrictive* (*p* = 0.002) and between *futurists* and *time restrictive* categories (*p* = 0.001).

**Table 3:** Analysis of Variance (ANOVA) or Kruskal-Wallis-Test for Differences between the four categories of persons possible Time Perspectives.

**Variable^b^**	**Time expansive^a^**	**Futurists^a^**	**Reminiscers^a^**	**Time restrictive^a^**	**F or *χ^2^***	***p^c^***
Delay of gratification1	3.08 (1.08)	3.17 (1.07)	2.40 (0.88)	1.96 (1.28)	17.61^**^	0.001
Delay of gratification2	2.68 (1.11)	2.87 (1.14)	2.2 (0.95)	1.81 (1.30)	10.68^*^	0.014
Delay discounting	−5.54 (2.01)	−6.05 (1.64)	−4.88 (0.89)	−5.15 (1.70)	6.96	0.073
Episodic future thinking, future	38.91 (10.65)	37.17 (10.33)	31.47 (8.96)	33.64 (7.64)	3.07^*^	0.032
Episodic future thinking, atemporal	39.98 (8.56)	36.55 (9.18)	33.56 (9.85)	34.88 (8.45)	2.01	0.118
Depression, overall	−0.51 (0.60)	−1.10 (0.72)	0.18 (1.05)	0.43 (1.23)	10.31^*^	0.016

*ANOVA and Kruskal-Wallis-Test respectively enclosed four different continuative Time Perspective categories. Persons scoring below the median on both the Past and Future orientation formed the time restrictive category. Persons scoring below the median on the Past but above on the Future orientation formed the futurist category. Participants scoring below the median on the Future but above on the Past orientation formed the reminiscers category and subjects scoring above the medians on both orientations formed the time expansive category*.

a *Unless otherwise specified, the data represent means (±SD)*.

b *Variables analyzed via ANOVA: Episodic Future Thinking, Future; Episodic Future Thinking, Atemporal. Variables analyzed via Kruskal-Wallis-Test: Delay of Gratification1 (built with the cutoff between 4 and 5 on the snack scale); Delay of Gratification2 (built with the cutoff between 6 and 7 on the snack scale); Delay Discounting*.

c *p-value Kruskal-Wallis-Test and ANOVA*.

* *p < 0.05*,

** *p < 0.01*.

The participants that could imagine future events and siutations the best (EFT-F) were people in the *time expansive* category (*M* = 38.91) followed by the category comprising *futurist* (*M* = 37.17), *time restrictive* (*M* = 33.64), and *reminiscers* (*M* = 31.47)*. Post-hoc* tests for EFT-F showed that the categories of *time restrictive* and *time expansive* differed significantly (*p* = 0.041). Besides, we could find significant differences between *reminiscers* and *futurists* (*p* = 0.045) as well as *reminiscers* and *time expansive* (*p* < 0.008).

Furthermore, we found significant age differences within the particular categories [$\chi_{\left(3,\;\text{N}\;=\;95\right)}^{2}$ = 9.58, *p* = 0.022]. The participants of the youngest age group (9–14 years) were mostly assigned to the *time expansive category* (7 vs. 3 in the *reminiscers* vs. 4 in the *futurists* vs. 4 in the *time restrictive* category). The members of the younger age group (18–24 years) mostly belonged to the *futurists* category (11 vs. 2 in the *reminiscers* vs. 9 in the *time restrictive* vs. 3 in the *time expansive* category). The participants belonging to the middle-aged group were represented mostly in the *time expansive* category (7 vs. 1 in the *reminiscers* vs. 5 *in the futurists* vs. 5 in the *time restrictive* category). The older participants of the sample were the strongest in the *reminiscers* category (9 vs. 5 in the *time expansive* vs. 3 in the *futurist* vs. 7 in the *time restrictive* category). Finally, the members of the oldest age group, similar to the older participants, were mostly represented in the *reminiscers* category (5 vs. 3 in the *time expansive* vs. 0 in the *futurists* vs. 2 in the *time restrictive* category).

A comparison of the z-scores of depression scores revealed significant differences between the categories [$\chi_{\left(3,\text{textitN}=94\right)}^{2}$ = 10.31, *p* = 0.016]. The participants of the *time expansive* category had the lowest depression scores (*M* = −0.51), followed by *futurists* (*M* = −0.10), *reminiscers* (*M* = 0.18) and *time restrictive* (*M* = 0.43).

## Discussion

We could show that there is an age difference in the ability to self-regulate. Middle-aged participants showed significantly higher self-regulation than the oldest participants. They also showed better performances than the youngest participants (see DD curve in Figure 1) but without significant differences. Furthermore, the results indicate that a FTP has a positive relation to the DoG and DD behavior, which applied for every age group in our sample. A closer examination of time perspective variables revealed that an FTP decreases with age, while a PTP increases with age. Furthermore, younger and older participants showed a weaker EFT-F than the middle-aged participants. Taken together, while the study replicated several recent findings on SR across the life span, these results also provide important new insights in the understanding of the development of self-regulatory behavior and its influencing factors. These insights are discussed in more detail below.

First, age differences for DoG could be found only with an alpha level of 10%. However, a significant effect of age on DD was observed, as expected. This implies that younger and older participants showed less self-controlled behavior than middle-aged participants.

The hypothesis about the association between DoG/DD and the future orientation constructs was confirmed for DoG/DD and FTP in all age groups. Besides, the analysis showed no significant associations between DoG/DD and PTP, as expected. Contrary to our expectations, the correlations between DoG and EFT-F/EFT-P and between DD and EFT-F/EFT-P were not significant independent of age.

The third hypothesis concerning the age differences with regard to the future orientation constructs could be confirmed. The findings revealed significant group differences for both constructs. While the data showed an inverse U-shaped form for EFT-F with the best performance from middle-aged participants, the older and oldest participants scored lower on the FTP measure. In contrast, PTP was significantly associated with age.

### Delay of gratification, delay discounting, and age

A strong relationship between DoG and DD was highlighted through the study to replicate the well-established finding that DoG and DD are very close constructs (e.g., Mischel et al., 1989; Evenden and Ryan, 1996). Furthermore, the results revealed an impact of age on DD. The five age groups showed different DD behavior, as expected. The younger and older participants showed a less self-controlled behavior than the middle-aged subjects (see Figure 1). The regression equation for the relationship between DD and age supports these predictions. It resulted in a U-shaped function with better performances for the middle-aged participants and weaker performances for the younger and older, as predicted. These findings concord with those of other studies. For example, Steinberg et al. (2009) highlighted that younger participants (10–15 years) show less SR in DD task than older participants (16–30 years). Several other studies also arrived at the same conclusion (e.g., Reimers et al., 2009). The crucial difference of our findings is that we could extend these findings by showing that older adults show a weaker SR, similar to younger people, and that middle-aged participants seem to be the most controlled.

As described in the introduction, young and old-aged individuals live with more uncertainties than the middle-aged individuals (Trostel and Taylor, 2001; Sozou and Seymour, 2003). Often, younger people have a late career start and spend significant time in their job training, without a guarantee of a job in the future. Additionally, the younger generation faces insecurities in their private life either, such as, choosing a life partner or choosing a city to reside. These insecurities about the future might be responsible for causing the prioritization of immediate before delayed gratification. Similarly, the older age groups also face insecurities, but in other areas of their life. For example, illness, loss of relatives and friends, and coming close to death make future rewards less attractive, thereby leading to impulsive and less long-term decisions. Green et al. (1999) also investigated the DD behavior across the life span (11–79 years). They came to the conclusion that SR increases with age. The findings of current study contradict their results and indicate a rise in SR from childhood to adulthood, and a decrease in self-controlled behavior after the middle adulthood age. The difference in the age span (11–79 years, Green et al. vs. 9–101 years, in our study) possibly accounts for differences in findings. There are possibilities that the wider sampling used in this study provided a more differentiable picture of SR across the life span.

The better fit of the quadratic function for the relationship between DD and age is also supported by the findings on education and income. The sample showed a linear increase of these two variables depending on age. Nevertheless, a quadratic function resulted in a better fit for the relationship between DD and age than a linear function did. Therefore, despite of a different linear pattern of age in the sample, the quadratic function emerges to be the best fit for the relationship between DD and age.

There might be questions on the lack of findings on these age differences for DoG, in this study, although a strong negative association between DD and DoG was established. One plausible explanation is provided by Metcalfe and Mischel (1999) and their two-system model. It postulates that processes underlying SR can be either *hot* or *cool*. The hot system is the affective and motivational part of the systems. In contrast, the cool system is rather cognitive, strategic and emotionally neutral. Zelazo and Carlson (2012) developed a very similar paradigm involving executive functions (EF) in the process of SR that distinguish between *hot* and *cool* too. The measures employed in the current study—real snacks, magazines and money rewards—to determine DoG seem to activate mainly the motivational-affective system (Baumeister and Vohs, 2004). In contrast, the DD questionnaire using hypothetical money rewards, and the inclusion of different intervals and amount of money, requires more cognitive resources. This is because the tasks involve mathematical operations instead of actual behavioral measurement like in the DoG tasks. As a consequence, DoG and DD might differ since the former can be assigned to the *hot* system, while DD can be assigned to the cool system (Hongwanishkul et al., 2005). In addition, there is evidence for age differences on EF, and in the same way, we expected age differences on SR. Therefore, it could be shown that younger (*M* = 8.8 years) and older age groups (*M* = 71.1 years) showed weaker performance in EF than a middle-aged group of participants (*M* = 22.3 years). The results indicated, as our analysis did for SR too, a quadratic curve for EF over the life span with a performance peak in middle age (Zelazo et al., 2004). Besides, different studies have already shown that cognitive functions develop in childhood and decrease with age (e.g., Deary et al., 2009). Therefore, performance in childhood and advanced age is worse than in the middle adulthood. This curve is consistent to our findings that DD is lowest (i.e., SR was highest) in young/middle-aged adults. In contrast, motivational processes seem to stay relatively stable till advanced age (Forstmeier and Maercker, 2015), which can explain the non-significant age differences in DoG and the comparatively high performance of the oldest age group.

An explanation for the high performance (see Table 1) of the youngest age group on DoG can be obtained from the average age of the children selected for the sample (*M* = 11.28; *SD* = 1.36). A majority of reviewed research concerning DoG behavior of children in comparison with adults used samples of children in the pre-school or elementary school age (e.g. Drobetz et al., 2012a, mean age of the participating children *M* = 6.00 years). However, DoG increases when children get older (Evans and Rosenbaum, 2008; Drobetz et al., 2012a), which naturally contributes toward an increase in their performances. Hence, it is concluded that the high performance of our youngest age group in the DoG task could be attributed to their age, which was comparatively higher than the age groups of children sampled in previous studies.

### Delay of gratification, delay discounting, and future orientations

The study also aimed to investigate the association between DoG and future orientations. The research hypothesized that an FTP could be positively correlated with a better performance in DoG. The results show that our hypothesis could be confirmed. Participants with a more distinctive FTP showed significantly higher DoG. This confirms studies in an academic context, which employ self-report measures and do not assess actual behavior like the current one (Bembenutty and Karabenick, 2004). Alternately, these studies investigate the relationship of similar constructs like the correlation between motivation/SR and personally valued future goals (Miller and Brickman, 2004). The current study showed for the first time that an FTP can be used with a behavioral measure, wherein the ability to wait for a bigger reward and deny an immediate smaller reward can be assessed.

In addition, the study could find this DoG–FTP relation across each of the five age groups. It implies that people who orient their lives toward the future can delay gratification more successfully than people with less future orientation, independent of their age. Furthermore, we found a significant negative relationship between FTP and age. It indicates that the younger and middle-aged participants had a stronger FTP, and this tendency decreased gradually in the older participants, and was at a minimum in the oldest age group. This result contradicts the findings of Steinberg et al. (2009), who found that individuals, particularly till the age of 16, expressed less concerns about the future and on the consequences of their decisions. This difference in finding can be attributed to the different ways of measuring future orientation. While the current study used the BTPS, Steinberg and colleagues used a self-report measure of future orientation that was especially developed for their study.

Furthermore, the difference in finding can also be attributed to the different age samples used in both studies. Steinberg and colleagues investigated the future orientation of a sample that was aged 30 years, at the most. These researchers might have also investigated a wider age span to establish a decrease in FTP with higher age. In our sample, BTPS was employed to reveal that participants between 9 and 55 years showed a similar increase in FTP when compared to the older age groups, where the perspective showed a decreasing tendency. In addition, the study found a positive significant relationship between a past time perspective and age. To sum it up, these results show that younger to middle-aged individuals tend to live with a stronger future orientation, while older persons are inclined to look into the past.

Similarly, the results show a significant negative relationship between FTP and DD, independent of age. A person with a future-oriented view seems to have a tendency toward making long-term decisions, and hence act in a less impulsive manner. This finding supports the strength of the effect between DoG and FTP, which is also established in the current study. The linkage between the constructs of FTP and DD seems to be logical, and, not surprisingly, previous literature has already dealt with its similarities and differences (Teuscher and Mitchel, 2011).

It is also important to mention another new finding concerning DoG and time perspective. On the basis of the BTPS (Webster, 2011), participants can be assigned to one of the four categories of time perspective (*time expansive, futurist, reminiscers*, and *time restrictive*). The *futurists—*with a strong future orientation and without past orientation—were the best in delaying gratification. People in the *time expansive category* were the participants that dealt most with their actions and with situations that either took place in the past or might occur in the future. These people were second best in delaying gratification, while people in the *time restrictive* and *reminiscers* category showed clearly lower performances (see Table 3). These results support the hypothesis that a time perspective that is directed toward the future or at least embraces one's future and past can promote the ability to delay gratification like already found for academic DoG by Bembenutty and Karabenick (2004).

Regarding the relationship between the BTPS categories and the age of the participants, we found significant differences and could replicate the findings of Webster and Ma (2013). Consistent with their results, the *futurist* category mostly included the young adults, while the older participants were strongly represented in the *reminiscers* category. These findings are supported by the correlations between age and the past or future orientation of the participants. The results go along with the outcomes of other studies, which indicated that the future orientation of individuals decreases with age (e.g., Lang and Carstensen, 2002), while the past orientation increases. In addition, we could confirm findings that postulated higher depression scores for individuals with an orientation rather directed into the past (e.g., Brandtstädter and Wentura, 1994). Our results also can be linked to different approaches regarding depression that connect depression to hopelessness. Negative thinking about the future or pessimism about the future is in turn a central symptom of hopelessness (Beck, 1967; Brown and Harris, 1978; Lavender and Watkins, 2004). The differences of the categories on EFT-F support a linkage to these approaches. The best time travelers were, like the participants with lower depression scores, in the categories with an orientation directed rather into future.

Furthermore, the study hypothesized that people with a better ability to imagine future scenarios, events, etc., make less impulsive choices and are more oriented toward the future. Several other studies also investigated the relationship between DD and EFT. These studies came to the conclusion that EFT tasks during a delay discounting performance reduce the discounting of delayed reward because EFT activates brain regions involved in prospective thinking (Peters and Büchel, 2010; Daniel et al., 2015). As already mentioned in the introduction, the expected relationship between DoG/DD and EFT can be easily understood. If people have a better ability to imagine future events or situations, including future rewards, then they are more likely to wait for gratification. This factor is attributed to the preciseness and alertness of imagination. Contrary to these considerations, the study could not find any relationships between DoG/DD and EFT. One plausible explanation is that we randomized the order of the DoG and EFT tasks. Contrary to Daniel et al. (2015) we presented the EFT task or the DoG task first alternately. Maybe there would have been significant correlations if we presented the EFT task first over the entire sample. A similar problem appears for the DD task. The half of the sample did not finish the DD task directly before finishing the EFT task. The procedure was interrupted through the DoG task. Therefore, maybe the EFT task did activate patterns of future thinking, but because of this interruption, they did not stay activated till the DD task, for the half of the participants.

### Future orientations and age

Furthermore, we established that people differ significantly in their ability to travel mentally into the future, depending on their age. The EFT-F performance of middle-aged participants repeated in this instance. The *post hoc* tests yielded that the middle-aged adults (35–55 years) performed significantly better than the other age groups except the oldest age group (but nevertheless better). These results were again supported by the regression equation calculated in the study, wherein the hypothesized inversed U-shaped function with weaker performances for younger and older participants could be confirmed. Several other studies already found that the ability to imagine new scenarios in the future decreases with age (Addis et al., 2008). Due to the factor that one can find an increase in EFT over the life span, from childhood to adolescence (Gott and Lah, 2014) established the constructive-episodic-simulation hypothesis, which postulates the need for a system that flexibly retransforms details from past events to imagine future episodes. This ability to retransform sometimes raises problems for older people because relational processes that combine parts of an episode work effectively only until young age (Lyle et al., 2006; Addis et al., 2008). Therefore, the loss in cognitive capacity is contributed to the weaker performance of the older participants (e.g., Rendell et al., 2012). The younger participants, on the other hand, probably could not develop their mental skills as much as the middle- aged group, and hence, they showed less ability to mentally travel into time.

## Limitations and further perspectives

A potential weakness of the study lies in the relatively small sample size per age group. Furthermore, the study did not test children aged 8 and below. A majority of research concerning children's DoG used samples of children that were younger than those in our sample. An additional age group with younger children might have led to significant age differences in DoG in an expected manner.

Besides, it is very unfavorable that most of the variables, and especially the variables that measure self-regulation, were not normally distributed. We had to conduct most of the analyses using non-parametric tests. Therefore, we did not have the possibility to include covariates in analyses with these variables. Because of the strong correlation between DoG and DD, an MANCOVA to analyze the age differences on these variables certainly would have made sense.

Another potential problem appears in the high correlation between EFT-F and EFT-A (*r* = 0.741, *p* < 0.01). The constructs seem to be harder to differentiate than expected. One reason for the high correlation may be that participants in both conditions were asked to develop new scenarios in established situations. In the EFT-F scenarios, they should additionally imagine themselves and the whole scenario in the future. Nevertheless, this instruction, and thus, the difference between the tasks may have been too weak. Therefore, prospective studies in the context of EFT should use measures and analysis methods respecting and weighting the future aspects more intense to gain a clearer differentiation between EFT and imagination vividness.

The cross-sectional design of our study exhibits one big advantage. The study can facilitate comparisons between different age groups and can draw a picture of DoG and its potential influences across the life span without having to collect data for 60 years. The disadvantage is that the data analysis only allows computation of correlations. This type of investigation does not facilitate casual conclusions. Nevertheless, we were able to compare the DoG behavior of a broad age span of participants. The used DoG test allowed us to test and compare DoG for every age group the same way because it used four types of reward. Therefore, it was suitable for every age group that was investigated in this study.

A more precise view of the DoG development over the life span can be obtained by testing the important variables in a longitudinal design. This would facilitate procurement of more reliable data while allowing investigation into causal effects of DoG and influencing variables. Further research should look into a sample that includes younger children in the analysis. Testing a wider age span can produce meaningful results and facilitate examination of additional differences that might arise from a wider sampling. We did not include younger children in our sample because the DoG test would not have been applicable to children younger than 9 years. Previous studies used other methods to test the DoG behavior of children in earlier childhood (e.g., marshmallow paradigm or similar modified versions, Mischel et al., 1989; Drobetz et al., 2012b). However, one of our main goals was to compare adult's and children's self-regulation using the same measure to prevent losing effects because of testing it in different ways for different age groups.

Taken together, the present study helps understanding how self-regulation develops over the life span and its influencing factors. Therefore, we could confirm a direct relationship between DoG and a time perspective that is directed into the future and that this perspective decreases with age. Furthermore, we showed that younger and older persons have difficulties with imagining the future when compared to middle-aged persons. These results might be of interest for other basic researchers that investigate the development of self-regulatory skills over the lifespan. They can help to get the bigger picture of understanding self-regulation and provide new starting points for further research. Additionally, there might be implications for intervention programs with the goal of practicing these self-regulatory skills not only in childhood and adolescence but also in advanced age. Programs could install training elements were not only self-regulation itself (e.g., learning that valued rewards can be obtained by effort, Strayhorn, 2002) is practiced but where also the advantages of an FTP are pointed out and trained. Furthermore, clinical intervention could benefit from our results. Forstmeier and Rüddel (2007) showed that volitional competences support the efficiency of psychotherapy. Trying to take a future time perspective could be part of practicing these volitional competences.

## Compliance with ethical standards

Ethical approval: All procedures performed in studies involving human participants were in accordance with the ethical standards of the institutional committee and with the 1964 Helsinki declaration and its later amendments or comparable ethical standards.

Informed consent: Informed consent was obtained from all individual participants included in the study (see Method-section).

## Author contributions

MK, SF, NB, and LG developed the concept of the study. SF and LG conceived of the study, led its design and coordination, and drafted the manuscript. MK and NB organized the rating of episodic future thinking, provided expertise and oversaw the overall methodology. All authors reviewed and commented on drafts of the protocol and paper. All authors read and approved the final manuscript.

### Conflict of interest statement

The authors declare that the research was conducted in the absence of any commercial or financial relationships that could be construed as a potential conflict of interest.

## Footnotes

^1^We computed an alternative score (DoG2) for the DoG behavior of all participants. During this calculation we used a different cutoff for the snack subscale. In contrast to the DoG1 score we now counted a participant as delayer when he or she chose to delay 7 of the 8 snacks minimum (the DoG1 score was built with a cutoff between 4 and 5 delaying decisions, which has been the midpoint of the 8 decisions. Forstmeier et al. (2011) also used this cutoff in the original article. We added an alternative cutoff to check for different results using a stronger distinction between delayer and non-delayer. As you can see, the different cutoffs did not lead to significantly different results. Other studies also used slightly modified versions of original measures; see Podjarny et al., 2017).
